# A Survey of Computational Tools to Analyze and Interpret Whole Exome Sequencing Data

**DOI:** 10.1155/2016/7983236

**Published:** 2016-12-14

**Authors:** Jennifer D. Hintzsche, William A. Robinson, Aik Choon Tan

**Affiliations:** ^1^Division of Medical Oncology, Department of Medicine, School of Medicine, Aurora, CO 80045, USA; ^2^University of Colorado Cancer Center, Aurora, CO 80045, USA; ^3^Department of Biostatistics and Informatics, Colorado School of Public Health, University of Colorado, Anschutz Medical Campus, Aurora, CO 80045, USA

## Abstract

Whole Exome Sequencing (WES) is the application of the next-generation technology to determine the variations in the exome and is becoming a standard approach in studying genetic variants in diseases. Understanding the exomes of individuals at single base resolution allows the identification of actionable mutations for disease treatment and management. WES technologies have shifted the bottleneck in experimental data production to computationally intensive informatics-based data analysis. Novel computational tools and methods have been developed to analyze and interpret WES data. Here, we review some of the current tools that are being used to analyze WES data. These tools range from the alignment of raw sequencing reads all the way to linking variants to actionable therapeutics. Strengths and weaknesses of each tool are discussed for the purpose of helping researchers make more informative decisions on selecting the best tools to analyze their WES data.

## 1. Introduction

Recent advances in next-generation sequencing technologies provide revolutionary opportunities to characterize the genomic landscapes of individuals at single base resolution for identifying actionable mutations for disease treatment and management [1, 2]. Whole Exome Sequencing (WES) is the application of the next-generation technology to determine the variations in the exome, that is, all coding regions of known genes in a genome. For example, more than 85% of disease-causing mutations in Mendelian diseases are found in the exome, and WES provides an unbiased approach to detect these variants in the era of personalized and precision medicine. Next-generation sequencing technologies have shifted the bottleneck in experimental data production to computationally intensive informatics-based data analysis. For example, the Exome Aggregation Consortium (ExAC) has assembled and reanalyzed WES data of 60,706 unrelated individuals from various disease-specific and population genetic studies [3]. To gain insights in WES, novel computational algorithms and bioinformatics methods represent a critical component in modern biomedical research to analyze and interpret these massive datasets.

**Figure 1:**
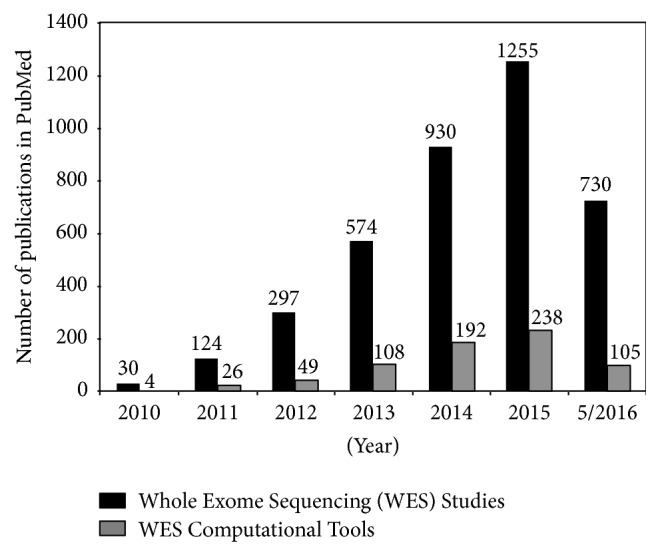
Trends in Whole Exome Sequencing studies and tools by querying PubMed (2011–2016).

**Figure 2:**
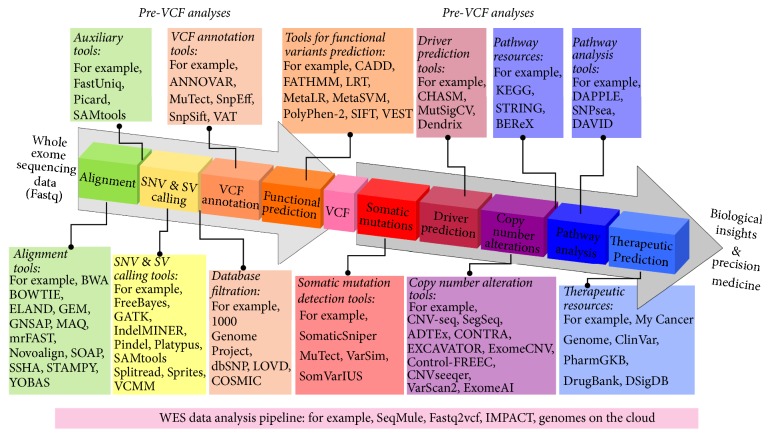
Whole Exome Sequencing data analysis steps. Novel computational methods and tools have been developed to analyze the full spectrum of WES data, translating raw fastq files to biological insights and precision medicine.

Genomic studies that employ WES have increased over the years, and new bioinformatics methods and computational tools have developed to assist the analysis and interpretation of this data (Figure 1). The majority of WES computational tools are centered on the generation of a Variant Calling Format (VCF) file from raw sequencing data. Once the VCF files have been generated, further downstream analyses can be performed by other computational methods. Therefore, in this review we have classified bioinformatics methods and computational tools into Pre-VCF and Post-VCF categories. Pre-VCF workflows include tools for aligning the raw sequencing reads to a reference genome, variant detection, and annotation. Post-VCF workflows include methods for somatic mutation detection, pathway analysis, copy number alterations, INDEL identification, and driver prediction. Depending on the nature of the hypothesis, beyond VCF analysis can also include methods that link variants to clinical data as well as potential therapeutics (Figure 2).

Computational tools developed to align raw sequencing data to an annotated VCF file have been well established. Most studies tend to follow workflows associated with GATK [4–6], SAMtools [7], or a combination of these. In general, workflows start with aligning WES reads to a reference genome and noting reads that vary. The most common of these variants are single nucleotide variants (SNVs) but also include insertions, deletions, and rearrangements. The location of these variants is used to annotate them to a specific gene. After annotation, the SNVs found can be compared to databases of SNVs found in other studies. This allows for the determination of frequency of a particular SNV in a given population. In some studies, such as those relating to cancer, rare somatic mutations are of interest. However, in Mendelian studies, the germline mutational landscape will be of more interest than somatic mutations. Before a final VCF file is produced for a given sample, software can be used to predict if the variant will be functionally damaging to the protein for prioritizing candidate genes for further study.

Bioinformatics methods developed beyond the establishment of annotated VCF files are far less established. In cancer research, the most established types of beyond VCF tools are focused on the detection of somatic mutations. However, there are strides being made to develop other computational tools including pathway analysis, copy number alteration, INDEL identification, driver mutation predictions, and linking candidate genes to clinical data and actionable targets.

Here, we will review recent computational tools in the analysis and interpretation of WES data, with special focus on the applications of these methods in cancer research. We have surveyed the current trends in next-generation sequencing analysis tools and compared their methodology so that researches can better determine which tools are the best for their WES study and the advancement of precision medicine. In addition, we include a list of publicly available bioinformatics and computational tools as a reference for WES studies (Table 1).

## 2. Computational Tools in Pre-VCF Analyses

Alignment, removal of duplicates, variant calling, annotation, filtration, and prediction are all parts of the steps leading up to the generation of a filtered and annotated VCF file. Here we review each one of these steps, as shown in Figure 2, and compare and contrast some of the tools that can be used to perform the Pre-VCF analysis steps.

### 2.1. Alignment Tools

The first step in any analysis of next-generation sequencing is to align the sequencing reads to a reference genome. The two most common reference genomes for humans currently are hg18 and hg19. Several aligning algorithms have been developed including but not limited to BWA [8], Bowtie 1 [9] and 2 [10], GEM [11], ELAND (Illumina, Inc.), GSNAP [12], MAQ [13], mrFAST [14], Novoalign (http://www.novocraft.com/), SOAP 1 [15] and 2 [16], SSAHA [17], Stampy [18], and YOABS [19]. Each method has its own unique features and many papers have reviewed the differences between them [20–22], and we will not review these tools in depth here. The three most commonly used of these algorithms are BWA, Bowtie (1 and 2), and SOAP (1 and 2).

### 2.2. Auxiliary Tools

Some auxiliary tools have been developed to filter aligned reads to ensure higher quality data for downstream analyses. PCR amplification can introduce duplicate reads of paired-end reads in sequencing data. These duplicate reads can influence the depth of the mapped reads and downstream analyses. For example, if a variant is detected in duplicate reads, the proportion of reads containing a variant could pass the threshold needed for variant calling, thus calling a falsely positive variant. Therefore, removing duplicate reads is a crucial step in accurately representing the sequencing depth during downstream analyses. Several tools have been developed to detect PCR duplicates including Picard (http://picard.sourceforge.net./), FastUniq [23], and SAMtools [7]. SAMtools rmdup finds reads that start and end at the same position, find the read with the highest quality score, and mark the rest of the duplicates for removal. Picard finds identical 5′ positions for both reads in a mate pair and marks them as duplicates. In contrast, FastUniq takes a* de novo* approach to quickly identify PCR duplicates. FastUniq imports all reads, sorts them according to their location, and then marks duplicates. This allows FastUniq not to require complete genome sequences as prerequisites. Due to the different algorithms each of these tools use, these tools can remove PCR duplicates individually or in combination.

### 2.3. Methods for Single Nucleotide Variants (SNVs) Calling

After sequences have been aligned to the reference genome, the next step is to perform variant detection in the WES data. There are four general categories of variant calling strategies: germline variants, somatic variants, copy number variations, and structural variants. Multiple tools that perform one or more of these variant calling techniques were recently compared to each other [24]. Some common SNV calling programs are GATK [4–6], SAMtools [7], and VCMM [25]. The actual SNV calling mechanisms of GATK and SAMtools are very similar. However, the context before and after SNV calling represents the differences between these tools. GATK assumes each sequencing error is independent while SAMtools believes a secondary error carries more weight. After SNV calling GATK learns from data while SAMtools relies on options of the user, Variant Caller with Multinomial probabilistic Model (VCMM) is another tool developed to detect SNVs and INDELs from WES and Whole Genome Sequencing (WGS) studies using a multinomial probabilistic model with quality score and a strand bias filter [25]. VCMM suppressed the false-positive and false-negative variant calls when compared to GATK and SAMtools. However, the number of variant calls was similar to previous studies. The comparison done by the authors of VCMM demonstrated that while all three methods call a large number of common SNVs, each tool also identifies SNVs not found by the other methods [25]. The ability of each method to call SNVs not found by the others should be taken into account when choosing a SNV variant calling tool(s).

### 2.4. Methods for Structural Variants (SVs) Identification

Structural Variants (SVs) such as insertions and deletions (INDELs) in high-throughput sequencing data are more challenging to identify than single nucleotide variants because they could involve an undefined number of nucleotides. The majority of WES studies follow SAMtools [7] or GATK [4–6] workflows which will identify INDELs in the data. However, other software has been developed to increase the sensitivity of INDEL discovery while simultaneously decreasing the false discovery rate.

Platypus [26] was developed to find SNVs, INDELs, and complex polymorphisms using local* de novo* assembly. When compared to SAMtools and GATK, Platypus had the lowest Fosmid false discovery rate for both SNVs and INDELs in whole genome sequencing of 15 samples. It also had the shortest runtime of these tools. However, GATK and SAMtools had lower Fosmid false discovery rates than Platypus when finding SNVs and INDELs in WES data [26]. Therefore, Platypus seems to be appropriate for whole genome sequencing but caution should be used when utilizing this tool with WES data.

FreeBayes uses a unique approach to INDEL detection compared to other tools. The method utilizes haplotype-based variant detection under a Bayesian statistics framework [27]. This method has been used in several studies in combination with other approaches for the identifying of unique INDELs [28, 29].

Pindel was one of the first programs developed to address the issue of unidentified large INDELs due to the short length of WGS reads [30]. In brief, after alignment of the reads to the reference genome, Pindel identifies reads where one end was mapped and the other was not [30]. Then, Pindel searches the reference genome for the unmapped portion of this read over a user defined area of the genome [30]. This split-read algorithm successfully identified large INDELs. Other computational tools developed after Pindel still utilize this algorithm as the foundation in their methods for detecting INDELs.

Splitread [31] was developed to specifically identify structural variants and INDELs in WES data from 1 bp to 1 Mbp building on the split-read approach of Pindel [30]. The algorithms used by SAMtools and GATK limit the size of structural variants, with variants greater than 15 bp rarely being identified [31]. Splitread anchors one end of a read and clusters the unanchored ends to identify size, content, and location of structural variants [31]. When compared to GATK, Splitread called 70% of the same INDELs but identified 19 more unique INDELs, 13 of which were verified by sanger sequencing [31]. The unique ability of Splitread to identify large structural variants and INDELs merits it being used in conjunction with other INDEL detecting software in WES analysis.

Recently developed indelMINER is a compilation of tools that takes the strengths of split-read and* de novo* assembly to determine INDELs from paired-end reads of WGS data [32]. Comparisons were done between SAMtools, Pindel, and indelMINER on a simulated dataset with 7,500 INDELs [32]. SAMtools found the least INDELs with 6,491, followed by Pindel with 7,239 and indelMINER with 7,365 INDELs identified. However, indelMINER's false-positive percentage (3.57%) was higher than SAMtools (2.65%) but lower than Pindel (4.53%). Conversely, indelMINER did have the lowest number of false-negatives with 398 compared to 589 and 1,181 for Pindel and SAMtools, respectively. Each of these tools has their own strengths and weaknesses as demonstrated by the authors of indelMINER [32]. Therefore, it can be predicted that future tools developed for SV detection will take an approach similar to indelMINER in trying to incorporate the best methods that have been developed thus far.

Most of the recent SV detection tools rely on realigning split-reads for detecting deletions. Instead of a more universal approach like indelMINER, Sprites [33] aims to solve the problem of deletions with microhomologies and deletions with microinsertions. Sprites algorithm realigns soft-clipping reads to find the longest prefix or suffix that has a match in the target sequence. In terms of the *F*-score, Sprites performed better than Pindel using real and simulated data [33].

All of these tools use different algorithms to address the problem of structural variants, which are common in human genomes. Each of these tools has strengths and weaknesses in detecting SVs. Therefore, it is suggested to use several of these tools in combination to detect SVs in WES.

### 2.5. VCF Annotation Methods

Once the variants are detected and called, the next step is to annotate these variants. The two most popular VCF annotation tools are ANNOVAR [34] and MuTect [35] which is part of the GATK pipeline. ANNOVAR was developed in 2010 with the aim to rapidly annotate millions of variants with ease and remains one of the popular variant annotation methods to date [34]. ANNOVAR can use gene, region, or filter-based annotation to access over 20 public databases for variants annotation. MuTect is another method that uses Bayesian classifiers for detecting and annotating variants [34, 35]. MuTect has been widely used in cancer genomics research, especially in The Cancer Genome Atlas projects. Other VCF annotation tools are SnpEff [36] and SnpSift [37]. SnpEff can perform annotation for multiple variants and SnpSift allows rapid detection of significant variants from the VCF files [37]. The Variant Annotation Tool (VAT) distinguishes itself from other annotation tools in one aspect by adding cloud computing capabilities [38]. VAT annotation occurs at the transcript level to determine whether all or only a subset of the transcript isoforms of a gene is affected. VAT is dynamic in that it also annotates Multiple Nucleotide Polymorphisms (MNPs) and can be used on more than just the human species.

### 2.6. Database and Resources for Variant Filtration

During the annotation process, many resources and databases could be used as filtering criteria for detecting novel variants from common polymorphisms. These databases score a variant by its minor allelic frequency (MAF) within a specific population or study. The need for filtration of variants based on this number is subject to the purpose of the study. For example, Mendelian studies would be interested in including common SNVs while cancer studies usually focus on rare variants found in less than 1% of the population. NCBI dbSNP database, established in 2001, is an evolving database containing both well-known and rare variants from many organisms [39]. dbSNP also contains additional information including disease association, genotype origin, and somatic and germline variant information [39].

The Leiden Open Variation Database (LOVD) developed in 2005 links its database to several other repositories so that the user can make comparisons and gain further information [40]. One of the most popular SNV databases was developed in 2010 from the 1000 Genomes Project that uses statistics from the sequencing of more than 1000 “healthy” people of all ethnicities [41]. This is especially helpful for cancer studies, as damaging mutations found in cancer are often very rare in a healthy population. Another database essential for cancer studies is the Catalogue of Somatic Mutations In Cancer (COSMIC) [42]. This database of somatic mutations found in cancer studies from almost 20,000 publications allows for identification of potentially important cancer-related variants. More recently, the Exome Aggregation Consortium (ExAC) has assembled and reanalyzed WES data of 60,706 unrelated individuals from various disease-specific and population genetic studies [3]. The ExAC web portal and data provide a resource for assessing the significance of variants detected in WES data [3].

### 2.7. Functional Predictors of Mutation

Besides knowing if a particular variant has been previously identified, researchers may also want to determine the effect of a variant. Many functional prediction tools have been developed that all vary slightly in their algorithms. While individual prediction software can be used, ANNOVAR provides users with scores from several different functional predictors including SIFT, PolyPhen-2, LRT, FATHMM, MetaSVM, MetaLR, VEST, and CADD [34].

SIFT determines if a variant is deleterious using PSI-BLAST to determine conservation of amino acids based on closely related sequence alignments [43]. PolyPhen-2 uses a pipeline involving eight sequence based methods and three structure based methods in order to determine if a mutation is benign, probably deleterious, or known to be deleterious [44]. The Likelihood Ratio Test (LRT) uses conservation between closely related species to determine a mutations functional impact [45]. When three genomes underwent analysis by SIFT, PolyPhen-2, and LRT, only 5% of all predicted deleterious mutations were agreed to be deleterious by all three methods [45]. Therefore, it has been shown that using multiple mutational predictors is necessary for detecting a wide range of deleterious SNVs. FATHMM employs sequence conservation within Hidden Markov Models for predicting the functional effects of protein missense mutation [46]. FATHMM weighs mutations based on their pathogenicity by the predicted interaction of the protein domain [46].

MetaSVM and MetaLR represent two ensemble methods that combine 10 predictor scores (SIFT, PolyPhen-2 HDIV, PolyPhen-2 HVAR, GERP++, MutationTaster, Mutation Assessor, FATHMM, LRT, SiPhy, and PhyloP) and the maximum frequency observed in the 1000 genomes populations for predicting the deleterious variants [47]. MetaSVM and MetaLR are based on the ensemble Support Vector Machine (SVM) and Logistic Regression (LR), respectively, for predicting the final variant scores [47].

The Variant Effect Scoring Tool (VEST) is similar to MetaSVM and MetaLR in that it uses a training set and machine learning to predict functionality of mutations [48]. The main difference in the VEST approach is that the training set and prediction methodology are specifically designed for Mendelian studies [48]. The Combined Annotation Dependent Depletion (CADD) method differentiates itself by integrating multiple variants with mutations that have survived natural selection as well as simulated mutations [49].

While all of these methods predict the functionality of a mutation, they all vary slightly in their methodological and biological assumptions. Dong et al. have recently tested the performance of these prediction algorithms on known datasets [47]. They pointed out that these methods rarely unanimously agree on if a mutation is deleterious. Therefore, it is important to consider the methodology of the predictor as well as the focus of the study when interpreting deleterious prediction results.

## 3. Computational Methods for Beyond VCF Analyses

After a VCF file has been generated, annotated, and filtered, there are several types of analyses that can be performed (Figure 2). Here we outline six major types of analyses that can be performed after the generation of a VCF file, with special focus on WES in cancer research: (i) significant somatic mutations, (ii) pathway analysis, (iii) copy number estimation, (iv) driver prediction, (v) linking variants to clinical information and actionable therapies, and (vi) emerging applications of WES in cancer research.

### 3.1. Methods to Determine Significant Somatic Mutations

After VCF annotation, a WES sample can have thousands of SNVs identified; however, most of them will be silent (synonymous) mutations and will not be meaningful for follow-up study. Therefore, it is important to identify significant somatic mutations from these variants. Several tools have been developed to do this task for the analysis of cancer WES data, including SomaticSniper [50], MuTect [35], VarSim [51], and SomVarIUS [52].

SomaticSniper is a computational program that compares the normal and tumor samples to find out which mutations are unique to the tumor sample, hence predicted as somatic mutations [50]. SomaticSniper uses the genotype likelihood model of MAQ (as implemented in SAMtools) and then calculates the probability that the tumor and normal genotypes are different. The probability is reported as a somatic score which is the Phred-scaled probability. SomaticSniper has been applied in various cancer research studies to detect significant somatic variants.

Another popular somatic mutation identification tool is MuTect [35], developed by the Broad Institute. MuTect, like SomaticSniper, uses paired normal and cancer samples as input for detecting somatic mutations. After removing low-quality reads, MuTect uses a variant detection statistic to determine if a variant is more probable than a sequencing error. MuTect then searches for six types of known sequencing artifacts and removes them. A panel of normal samples as well as the dbSNP database is used for comparison to remove common polymorphisms. By doing this, the number of somatic mutations is not only identified but also reduced to a more probable set of candidate genes. MuTect has been widely used in Broad Institute cancer genomics studies.

While SomaticSniper and MuTect require data from both paired cancer and normal samples, VarSim [51] and SomVarIUS [52] do not require a normal sample to call somatic mutations. Unlike most programs of its kind, VarSim [51] uses a two-step process utilizing both simulation and experimental data for assessing alignment and variant calling accuracy. In the first step, VarSim simulates diploid genomes with germline and somatic mutations based on a realistic model that includes SNVs and SVs. In the second step, VarSim performs somatic variant detection using the simulated data and validates the cancer mutations in the tumor VCF. SomVarIUS is another recent computational method to detect somatic variants in cancer exomes without a normal paired sample [52]. In brief, SomVarIUS consists of 3 steps for somatic variant detection. SomVarIUS first prioritizes potential variant sites, estimates the probability of a sequencing error followed by the probability that an observed variant is germline or somatic. In samples with greater than 150x coverage, SomVarIUS identifies somatic variants with at least 67.7% precision and 64.6% recall rates, when compared with paired-tissue somatic variant calls in real tumor samples [52]. Both VarSim and SomVarIUS will be useful for cancer samples that lack the corresponding normal samples for somatic variant detection.

### 3.2. Computational Tools for Estimating Copy Number Alteration

One active research area in WES data analysis is the development of computational methods for estimating copy number alterations (CNAs). Many tools have been developed for estimating CNAs from WES data based on paired normal-tumor samples such as CNV-seq [53], SegSeq [54], ADTEx [55], CONTRA [56], EXCAVATOR [57], ExomeCNV [58], Control-FREEC (control-FREE Copy number caller) [59], and CNVseeqer [60]. For example, VarScan2 [61] is a computational tool that can estimate somatic mutations and CNAs from paired normal-tumor samples. VarScan2 utilizes a normal sample to find Somatic CNAs (SCNAs) by first comparing Q20 read depths between normal and tumor samples and normalizes them based on the amount of input data for each sample [61]. Copy number alteration is inferred from the log_2_ of the ratio of tumor depth to normal depth for each region [61]. Lastly, the circular segmentation (CBS) algorithm [62] is utilized to merge adjacent segments to call a set of SCNAs. These SCNAs could be further classified as large-scale (>25% of chromosome arm) or focal (<25%) events in the WES data [63].

Recently, ExomeAI was developed to detect Allelic Imbalance (AI) from WES data [64]. Utilizing heterozygous sites, ExomeAI finds deviations from the expected 1 : 1 ratio between an A- and B-allele in multiple tumor samples without a normal comparison. Absolute deviation of B-allele frequency from .05 is calculated and similar to VarScan2; the CBS algorithm is applied to each chromosomal arm [62]. In order to reduce the number of false positives, a database was created with 500 (and counting) normal samples to filter out known AIs. This represents a novel tool to analyze WES for the detection of recurrent AI events without matched normal samples.

A systematic evaluation of somatic copy number estimation tools for WES data has been recently published [63]. In this study, six computational tools for CNAs detection (ADTEx, CONTRA, Control-FREEC, EXCAVATOR, ExomeCNV, and VarScan2) were evaluated using WES data from three TCGA datasets. Using a SNP array as the reference, this study found that these algorithms gave highly variable results. The authors found that ADTEx and EXCAVATOR had the best performance with relatively high precision and sensitivity when compared to the reference set. The study showed that the current CNA detection tools for WES data still have limitations and called for more robust algorithms for this challenging task.

### 3.3. Computational Tools for Predicting Drivers in Cancer Exomes

Cancer is a disease driven by genetic variations and copy number alterations. These genetic events can be classified into two classes, “driver” and “passenger” mutations. Driver mutations are the key mutation that drive the development of cancer and provide a survival advantage, whereas passenger mutations are “by-stander” alterations that happen to be altered in the primary cells but do not provide a survival advantage. As the cancer exomes tend to have high mutational burdens, identifying the “driver” mutations from the “passenger” mutations is one of the key analyses in cancer research. Several tools have been developed to find driver mutations including but not limited to CHASM [65], Dendrix [66], and MutSigCV [67].

CHASM (Cancer-specific High-throughput Annotation of Somatic Mutations) uses random forest as the machine learning approach to distinguish the difference between driver and passenger mutations in cancer [65]. CHASM was trained on the curated driver mutations obtained from the COSMIC database (“positive examples”) and synthetic passenger mutations generated according to the background of base substitution frequencies observed in specific tumor types (“negative examples”). CHASM can achieve high sensitivity and specificity when discriminating between known driver missense mutations and randomly generated missense mutations when tested in real tumor samples. This method has been one of the popular driver detection prediction tools for cancer researchers and has been applied in various cancer genomic studies.

Another common driver mutation tool is MutSigCV developed to resolve the problem of extensive false-positive findings that overshadow true driver mutations [67]. As the size of cancer genomes sequenced has increased implausible genes (such as* TTN*) have been falsely reported as being related to cancer when in fact their large size just makes the probability they would be mutated by chance increase [67]. MutSigCV takes into account patient-specific mutation frequency and spectrum as well as gene-specific background mutation rates, expression level, and replication time. By pooling all of this available data into one tool, MutSigCV has become a standard tool used for driver mutation identification in cancer studies.

*De novo* Driver Exclusivity (Dendrix) is a novel computational tool to determine* de novo* driver pathways (gene sets) from somatic mutations in patient data [66]. The main goal of the Dendrix algorithm is to find gene sets with high coverage and high exclusivity properties from the somatic data. The high coverage property assumes most patients have at least one driver mutation in the gene set, whereas the high exclusivity property assumes that these driver mutations are rarely mutated together in the same patient. Two algorithms were developed in Dendrix, one based on a greedy algorithm and one based on the Markov Chain Monte Carlo (MCMC) algorithm, to measure sets of genes that exhibit both criteria. When Dendrix was applied to the TCGA data, the algorithms identified groups of genes that were mutated in large subsets of patients and these mutations were mutually exclusive. This tool provides an opportunity to analyze WES data to identify driver pathways in cancer genomic studies.

### 3.4. Methods for Pathway Analysis

After candidate somatic mutations have been identified; one common type of analysis is to determine which pathways are affected by these mutations. Common pathway resources and tools used for these types of analysis include KEGG [68], DAVID [69], STRING [70], BEReX [71], DAPPLE [72], and SNPsea [73].

KEGG represents one of the most popular databases for pathway analysis. DAVID is a popular online tool for performing functional enrichment analysis based on user defined gene lists. STRING is the largest protein-protein interactions database for querying and searching for interactions between user defined gene lists. BEReX integrates STRING, KEGG, and other data sources to explore biomedical interactions between genes, drugs, pathways, and diseases. Both STRING and BEReX allow users to perform functional enrichment analysis and the flexibility to explore the interactions between user defined gene lists by expanding the networks.

DAPPLE (Disease Association Protein-Protein Link Evaluator) uses literature reported protein-protein interactions to identify significant physical connectivity among the genes of interest [72]. DAPPLE hypothesizes that genetic variation affects underlying mechanisms only detectable by protein-protein interactions [72]. SNPsea is another pathway analysis tool that requires specific SNP data [73]. SNPsea calculates linkage disequilibrium between involved genes and uses a sampling approach to determine conditions that are affected by these interactions.

### 3.5. Computational Tools for Linking Variants to Treatments

The ability to link variants with actionable drug targets is an emerging research topic in precision medicine. Databases such as My Cancer Genome have provided the framework for these studies (https://www.mycancergenome.org/). My Cancer Genome provides a bridge between genomic data and clinical therapeutic treatments. Similarly, ClinVar provides information on the relationship between variants and clinical therapy [74]. By collecting both the variants and the clinical significance related to these variants, ClinVar offers a database for researchers to explore the significance of sequencing findings in the clinical setting [74]. Pharmacological databases such as PharmGKB [75], DrugBank [76], and DSigDB [77] provide the link between drug and drug targets (variants). For example, by querying a list of variants to one of these databases, it allows users to identify actionable targets via enrichment analysis for the repurposing of drugs.

Similarly, the ability to incorporate clinical data into sequencing studies is vital to the advancement of personalized medicine. However, due to the lack of integration between electronic health records (EHR) and molecular analysis, this remains one of the challenges in translating WES data analysis into clinical practice. Projects such as cBioPortal provide a framework for incorporating sequencing data with available clinical data [78]. New methods for addressing this task are urgently needed to take advantage of the important applications of WES data within the clinic in order to advance precision medicine.

## 4. WES Analysis Pipelines

WES data analysis pipelines integrate computational tools and methods described in the previous sections in a single analysis workflow. Here, we review three recent sequencing pipelines SeqMule [79], Fastq2vcf [80], and IMPACT [81] that assimilate some of the tools described in previous sections.

SeqMule stands out in part due to the use of five alignment tools (BWA, Bowtie 1 and 2, SOAP2, and SNAP) and five different variant calling algorithms (GATK, SAMtools, VarScan2, FreeBayes, and SOAPsnp) [79]. SeqMule contains at least one feature that performs Pre-VCF analyses to generate a filtered VCF file. SeqMule also generates an accompanying HTML-based report and images to show an overview of every step in the pipeline. Fastq2vcf also performs the Pre-VCF analyses using BWA as an alignment tool and variant calling by GATK, UnifiedGenotyper, HaplotypeCaller, SAMtools, and SNVer resulting in a filtered VCF after implementation of ANNOVAR and VEP [80]. Fastq2vcf can be used in a single or parallel computing environment on variety of sequencing data.

Both SeqMule and Fastq2vcf pipelines focus on taking raw sequencing data and converting it into a filtered VCF file. IMPACT (Integrating Molecular Profiles with ACtionable Therapeutics) WES data analysis pipeline was developed to take this analysis a step further by linking a filtered VCF to actionable therapeutics [81]. The IMPACT pipeline contains four analytical modules: detecting somatic variants; calling copy number alterations; predicting drugs against the deleterious variants; and tumor heterogeneity analysis. IMPACT has been applied to longitudinal samples obtained from a melanoma patient and identified novel acquired resistance mutations to treatment. IMPACT analysis revealed loss of CDKN2A as a novel resistance mechanism to the combination of dabrafenib and trametinib treatment and predicted potential drugs for further pharmacological and biological studies [81].

To compare the strengths and weaknesses between these three WES pipelines, SeqMule allows the use of different alignment algorithms in its pipeline whereas IMPACT and Fastq2vcf only utilize BWA as the sequencing alignment algorithm. SAMtools is the common tool used by IMPACT, Fastq2vcf, and SeqMule to call variants. In addition, Fastq2vcf and SeqMule employ GATK and other variant calling algorithms for variants detection. Fastq2vcf and IMPACT both annotate the variants with ANNOVAR. Fastq2vcf also utilizes VEP and IMPACT utilizes SIFT and PolyPhen-2 as the primary variants prediction methods. For Post-VCF analysis, IMPACT pipeline has more options as compared to SeqMule and Fastq2vcf. In particular, IMPACT performs copy number analysis, tumor heterogeneity, and linking of actionable therapeutics to the molecular profiles. However, IMPACT is only designed to be performed on tumor samples while SeqMule and Fastq2vcf are designed for any WES dataset. Therefore, it is advisable for the users to consider the analytic needs to select the appropriate WES data analysis pipeline for their research.

As recently discussed by Altman et al., part of the U.S. Precision Medicine Initiative (PMI) includes being able to define a gold standard of pipelines and tools for specific sequencing studies to enable a new era of medicine [82]. Automated pipelines such as these will accelerate the analysis and interpretation of WES data. Future development of data analysis pipeline will be needed to incorporate newer and wider tools tailored for specific research questions.

## 5. Conclusions

In summary, we have reviewed several computational tools for the analysis and interpretation of WES data. These computational methods were developed to generate VCF files from raw sequencing data, as well as tools that perform downstream analyses in WES studies. Each tool has specific strengths and weaknesses, and it appears that using several of them in combination would lead to more accurate results. Currently, there are still challenges for bioinformaticians at every step in analyzing WES data. However, the greatest area of need is in the development of tools that can link the information found in a VCF file to clinical databases and therapeutics. Research in this area will help to advance precision medicine by providing user-friendly and informative knowledge to transcend the laboratory.

## Figures and Tables

**Table 1:** 

Computational tools	Description	Website	References
*Alignment tools*			
Burrows-Wheeler Aligner (BWA)	Perform short reads alignment using BWT approach against a references genome allowing for gaps/mismatches.	http://bio-bwa.sourceforge.net/	[8]
Bowtie (1 & 2)	Performs short read alignment using the Burrows-Wheeler index in order to be memory efficient, while still maintaining an alignment speed of over 25 million 35 bp reads per hour.	http://bowtie-bio.sourceforge.net/index.shtml	[9, 10]
ELAND	Short read aligner that achieves speed by splitting reads into equal lengths and applying seed templates to guarantee hits with only 2 mismatches.	http://www.illumina.com/	Illumina, Inc.
GEM	Short read aligner using string matching instead of BWT to deliver precision and speed.	http://algorithms.cnag.cat/wiki/The_GEM_library	[11]
GSNAP	Performs short and long read alignment, detects long and short distance splicing, SNPs, and is capable of detecting bisulfite-treated DNA for methylation studies.	http://research-pub.gene.com/gmap/	[12]
MAQ	Short read aligner compatible with Illumina-Solexa and ABI SOLiD data, performs ungapped alignment allowing 2-3 mismatches for single-end reads and one mismatch for paired-end reads.	http://maq.sourceforge.net/	[13]
mrFAST	Performs short read alignment allowing for INDELs up to 8 bp, for Illumina generated data. Paired-end mapping using a one end anchored algorithm allows for detection of novel insertions.	http://mrfast.sourceforge.net/	[14]
Novoalign	Alignment done on paired-end or single-end sequences, also capable of doing methylation studies. Allows for a mismatch up to 50% of a read length and has built-in adapter and base quality trimming.	http://www.novocraft.com/products/novoalign/	http://www.novocraft.com/
SOAP (1 & 2)	SOAP2 improved speed by an order of magnitude over SOAP1 and can align a wide range of read lengths at the speed of 2 minutes for one million single-end reads using a two-way BWT algorithm.	http://soap.genomics.org.cn/	[15, 16]
SSAHA	Uses a hashing algorithm to find exact or close to exact matching in DNA and protein databases, analogous to doing a BLAST search for each read.	https://www.vectorbase.org/glossary/ssaha-sequence-search-and-alignment-hashing-algorithm/	[17]
Stampy	Alignment done using a hashing algorithm and statistical model, to align Illumina reads for genome, RNA, and Chip sequencing allowing for a large number or variations including insertions and deletions.	http://www.well.ox.ac.uk/project-stampy	[18]
YOABS	Uses a 0(*n*) algorithm that uses both hash and tri-based methods that are effective in aligning sequences over 200 bp with 3 times less memory and ten times faster than SSAHA.	Available by request for noncommercial use	[19]
HTSeq	Python based package with many functions to facilitate several aspects of sequencing studies.	http://www-huber.embl.de/HTSeq/doc/overview.html	

*Auxiliary tools*			
FastUniq	Imports, sorts, and identifies PCR duplicates of short sequences from sequencing data.	https://sourceforge.net/projects/fastuniq/	[23]
Picard	Picard is a set of command line tools for manipulating high-throughput sequencing (HTS) data and formats such as SAM/BAM/CRAM and VCF.	http://picard.sourceforge.net/	
SAMtools	Suite of tools capable of viewing, indexing, editing, writing, and reading SAM, BAM, and CRAM formatted files.	http://www.htslib.org/	[7]

*SNV and SV calling*			
GATK	Variant calling of SNPs and small INDELs; can also be used on nonhuman and nondiploid organisms.	https://www.broadinstitute.org/gatk/	[4–6]
SAMtools	Suite of tools capable of viewing, indexing, editing, writing, and reading SAM, BAM, and CRAM formatted files.	http://www.htslib.org/	[7]
VCMM	Detection of SNVs and INDELs using the multinomial probabilistic method in WES and WGS data.	http://emu.src.riken.jp/VCMM/	[25]
FreeBayes	Detection of SNPs, MNPs, INDELs, and structural variants (SVs) from sequencing alignments using Bayesian statistical methods.	https://github.com/ekg/freebayes	[27]
indelMINER	Splitread algorithm to identify breakpoint in INDELs from paired-end sequencing data.	https://github.com/aakrosh/indelMINER	[32]
Pindel	Detection of INDELs using a pattern growth approach with anchor points to provide nucleotide-level resolution.	http://gmt.genome.wustl.edu/packages/pindel/	[30]
Platypus	Detection of SNPs, MNPs, INDELs, replacements, and structural variants (SVs) from sequencing alignments using local realignment and local assembly to achieve high specificity and sensitivity.	http://www.well.ox.ac.uk/platypus	[26]
Splitread	Detection of INDELs less than 50 bp long from WES or WGS data, using a split-read algorithm.	http://splitread.sourceforge.net/	[31]
Sprites	Detection of INDELs is done using a split-read and soft-clipping approach that is especially sensitive in datasets with low coverage.	https://github.com/zhangzhen/sprites	[33]

*VCF annotation*			
ANNOVAR	Provides up-to-date annotation of VCF files by gene, region, and filters from several other databases.	http://annovar.openbioinformatics.org/	[34]
MuTect	Postprocesses variants to eliminate artifacts from hybrid capture, short read alignment, and next-generation sequencing.	http://www.broadinstitute.org/cancer/cga/mutect	[35]
SnpEff	Uses 38,000 genomes to predict and annotate the effects of variants on genes.	http://snpeff.sourceforge.net/	[36]
SnpSift	Tools to manipulate VCF files including filtering, annotation, case controls, transition, and transversion rates and more.	http://snpeff.sourceforge.net/SnpSift.html	[37]
VAT	Annotation of variants by functionality in a cloud computing environment.	http://vat.gersteinlab.org/	[38]

*Database filtration*			
1000 Genomes Project	Genotype information from a population of 1000 healthy individuals.	http://www.1000genomes.org/	[41]
dbSNP	Database of genomic variants from 53 organisms.	https://www.ncbi.nlm.nih.gov/projects/SNP/	[39]
LOVD	Open source database of freely available gene-centered collection of DNA variants and storage of patient and NGS data.	http://www.lovd.nl/3.0/home	[40]
COSMIC	Database containing somatic mutations from human cancers separated into expert curated data and genome-wide screen published in scientific literature.	http://cancer.sanger.ac.uk/cosmic	[42]
NHLBI GO Exome Sequencing Project (ESP)	Database of genes and mechanisms that contribute to blood, lung, and heart disorders through NGS data in various populations.	http://evs.gs.washington.edu/EVS/	
Exome Aggregation Consortium (ExAC)	Database of 60,706 unrelated individuals from disease and population exome sequencing studies.	http://exac.broadinstitute.org/	[3]
SeattleSeq Annotation	Part of the NHBLI sequencing project; this database contains novel and known SNVs and INDELs including accession number, function of the variant, and HapMap frequencies, clinical association, and PolyPhen predictions.	http://snp.gs.washington.edu/SeattleSeqAnnotation137/	

*Functional predictors*			
CADD	Machine learning algorithm to score all possible 8.6 million substitutions in the human reference genome from 1 to 99 based on known and simulated functional variants.	http://cadd.gs.washington.edu/info	[49]
FATHMM	Uses Hidden Markov Models to predict the functional consequences of SNVs in coding and noncoding variants through a web server.	http://fathmm.biocompute.org.uk/	[46]
LRT	Uses the Likelihood Ratio statistical test to compare a variant to known variants and determine if they are predicted to be benign, deleterious, or unknown.	http://genome.cshlp.org/content/19/9/1553.long	[45]
PolyPhen-2	Predicts potential impact of a nonsynonymous variant using comparative and physical characteristics.	http://genetics.bwh.harvard.edu/pph2/	[44]
SIFT	By using PSI-BLAST, a prediction can be made on the effect of a nonsynonymous mutation within a protein.	http://sift.jcvi.org/	[43]
VEST	Machine learning approach to determine the probability that a missense mutation will impair the functionality of a protein.	http://karchinlab.org/apps/appVest.html	[48]
MetaSVM & MetaLR	Integration of a Support Vector Machine and Logistic Regression to integrate nine deleterious prediction scores of missense mutations.	https://sites.google.com/site/jpopgen/dbNSFP	[47]

*Significant somatic mutations*			
SomaticSniper	Using two bam files as input, this tool uses the genotype likelihood model of MAZ to calculate the probability that the tumor and normal samples are different, thus identifying somatic variants.	http://gmt.genome.wustl.edu/packages/somatic-sniper/	[50]
MuTect	Using statistical analysis to predict the likelihood of a somatic mutation using two Bayesian approaches.	https://www.broadinstitute.org/cancer/cga/mutect	[35]
VarSim	By leveraging on previously reported mutations, a random mutation simulation is preformed to predict somatic mutations.	http://bioinform.github.io/varsim/	[51]
SomVarIUS	Identification of somatic variants from unpaired tissue samples with a sequencing depth of 150x and 67% precision, implemented in Python.	https://github.com/kylessmith/SomVarIUS	[52]

*Copy number alteration*			
Control-FREEC	Detects copy number changes and loss of heterozygosity (LOH) from paired SAM/BAM files by computing and normalizing copy number and beta allele frequency.	http://bioinfo-out.curie.fr/projects/freec/	[59]
CNV-seq	Mapped read count is calculated over a sliding window in Perl and R to determine copy number from HTS studies.	http://tiger.dbs.nus.edu.sg/cnv-seq/	[53]
SegSeq	Using 14 million aligned sequence reads from cancer cell lines, equal copy number alterations are calculated from sequencing data.	https://www.broadinstitute.org/cancer/cga/segseq	[54]
VarScan2	Determines copy number changes in matched or unmatched samples using read ratios and then postprocessed with a circular binary segmentation algorithm.	http://dkoboldt.github.io/varscan/using-varscan.html	[61]
ExomeAI	Detects allele imbalance including LOH in unmatched tumor samples using a statistical approach that is capable of handling low-quality datasets.	http://gqinnovationcenter.com/index.aspx	[64]
CNVseeqer	Exon coverage between matched sequences was calculated using log_2_⁡ ratios followed by the circular binary segmentation algorithm.	http://icb.med.cornell.edu/wiki/index.php?title=Elementolab/CNVseeqer&redirect=no	[60]
EXCAVATOR	Detects copy number variants from WES data in 3 steps using a Hidden Markov Model algorithm.	https://sourceforge.net/projects/excavatortool/	[57]
ExomeCNV	R package used to detect copy number variants of loss of heterozygosity from WES data.	https://secure.genome.ucla.edu/index.php/ExomeCNV_User_Guide	[58]
ADTEx	Detection of aberrations in tumor exomes by detecting B-allele frequencies and implemented in R.	http://adtex.sourceforge.net/	[55]
CONTRA	Uses normalized depth of coverage to detect copy number changes from targeted resequencing data including WES.	https://sourceforge.net/projects/contra-cnv/	[56]

*Driver prediction tools*			
CHASM	Machine learning method that predicts the functional significance of somatic mutations.	http://karchinlab.org/apps/appChasm.html	[65]
Dendrix	*De novo* drivers are discovered from cancer only mutational data including genes, nucleotides, or domains that have high exclusivity and coverage.	http://compbio.cs.brown.edu/projects/dendrix/	[66]
MutSigCV	Gene-specific and patient-specific mutation frequencies are incorporated to find mutations in genes that are mutated more often than would be expected by chance.	http://www.broadinstitute.org/cancer/software/genepattern/modules/docs/MutSigCV	[67]

*Pathway analysis tools and resources*			
KEGG	Database using maps of known biological processes that allows searching for genes and color coding of results.	http://www.genome.jp/kegg/	[68]
DAVID	Allows for users to input a large set of genes and discover the functional annotation of the gene list including pathways, gene ontology terms, and more.	https://david.ncifcrf.gov/	[69]
STRING	Network visualization of protein-protein interactions of over 2,031 organisms.	http://string-db.org/	[70]
BEReX	Uses biomedical knowledge to allow users to search for relationships between biomedical entities.	http://infos.korea.ac.kr/berex/	[71]
DAPPLE	Uses a list of genes to determine physical connectivity among proteins according to protein-protein interactions.	http://journals.plos.org/plosgenetics/article?id=10.1371/journal.pgen.1001273	[72]
SNPsea	Uses a linkage disequilibrium to determine pathways and cell types that are likely to be affected based on SNP data.	http://www.broadinstitute.org/mpg/snpsea/	[73]

*Tools and resources for linking variants to therapeutics*			
cBioPortal	Database that allows the download, analysis, and visualization of cancer sequencing studies, including providing patient and clinical data for samples.	http://www.cbioportal.org/	[78]
My Cancer Genome	Database for cancer research that provides linkage of mutational status to therapies and available clinical trials.	https://www.mycancergenome.org/	http://www.mycancergenome.org/
ClinVar	Database of relationship between phenotypes and human variations, showing the relationship between health status and human variations and known implications.	https://www.ncbi.nlm.nih.gov/clinvar/	[74]
DSigDB	Database of drug signatures that includes 19,531 genes and 17,389 compounds that can in part help identify compounds for drug repurposing studies in translational research.	http://tanlab.ucdenver.edu/DSigDB	[77]
PharmGKB	Knowledge base allowing visualization of a variety of drug-gene knowledge.	https://www.pharmgkb.org/	[75]
DrugBank	Contains detailed drug information with comprehensive drug target information for 8,206 drugs.	http://www.drugbank.ca/	[76]

*WES data analysis pipelines*			
fast2VCF	Whole Exome Sequencing pipeline that starts with raw sequencing (fastq) files and ends with a VCF file that has good capability for novel and expert users.	http://fastq2vcf.sourceforge.net/	[80]
SeqMule	WES or WGS pipeline that combines the information from over ten alignment and analysis tools to arrive at a VCF file that can be used in both Mendelian and cancer studies.	http://seqmule.openbioinformatics.org/en/latest/	[79]
IMPACT	WES data analysis pipeline that starts with raw sequencing reads and analyzes SNVs and CNAs and links this data to a list of prioritized drugs from clinical trials and DSigDB.	http://tanlab.ucdenver.edu/IMPACT/	[81]
Genomes on the Cloud (GotCloud)	Automated sequencing pipeline that performs in part alignment, variant calling, and quality control that can be run on Amazon Web Services EC2 as well as local machines and clusters.	http://genome.sph.umich.edu/wiki/GotCloud	
